# Total flavonoid– and nerve growth factor–loaded gelatin–genipin hydrogel improves repair after spinal cord injury

**DOI:** 10.4103/NRR.NRR-D-24-01445

**Published:** 2025-08-13

**Authors:** Yu Liang, Deshuang Xi, Yilin Teng, Pan Liu, Yanbing Feng, Qiumei Huang, Ming Gao, Shaohui Zong

**Affiliations:** 1 Department of Spine Osteopathia, The First Affiliated Hospital of Guangxi Medical University, Nanning, Guangxi Zhuang Autonomous Region, China; 2 Department of Spine Surgery, The Third Affiliated Hospital of Guangxi Medical University, Nanning, Guangxi Zhuang Autonomous Region, China; 3 Department of Orthopedics, The Third Affiliated Hospital of Xinxiang Medical University, Xinxiang, Henan Province, China; 4 Department of Spine Surgery, The Second Hospital of Shandong University, Jinan, Shandong Province, China; 5 Department of Anesthesiology, The Third Affiliated Hospital of Guangxi Medical University, Nanning, Guangxi Zhuang Autonomous Region, China; 6 Life Sciences Institute, Guangxi Medical University, Nanning, Guangxi Zhuang Autonomous Region, China; 7 Department of Orthopedics, Wuming Hospital of Guangxi Medical University, Nanning, Guangxi Zhuang Autonomous Region, China

**Keywords:** composited hydrogel, hawthorn leaf total flavonoids, nuclear factor kappa-B pathway, photothermal effect, reactive oxygen species, spinal cord injury

## Abstract

Rat nerve growth factor and total flavonoids from hawthorn leaf contribute to the recovery of neurological function after spinal cord injury, including traumatic, non-traumatic spinal cord injuries. However, it remains challenging to efficiently deliver nerve growth factor and total flavonoids from hawthorn leaf to spinal cord injury sites, ensure their sustained release, and minimize further damage. In the present study, we chose a biocompatible and biodegradable gelatin as the substrate, which was crosslinked with the natural biological crosslinker genipin to form a gelatin–genipin hydrogel carrier for the slow release of nerve growth factor and total flavonoids from hawthorn leaf in spinal cord injury sites. The prepared gelatin–genipin hydrogel had good injectable properties and photothermal effects. Furthermore, when the hydrogel with 2% genipin, 200 ng/mL nerve growth factor, and 320 μg/mL total flavonoids from hawthorn leaf was combined with near infrared irradiation, there was a slow release of total flavonoids from hawthorn leaf and nerve growth factor, reduced oxidative stress, an improved inflammatory microenvironment, and accelerated angiogenesis and axonal regeneration via inhibition of the nuclear factor kappa-B signaling pathway, thereby promoting recovery from spinal cord injury. Collectively, our results indicate that this new hydrogel may improve the prognosis of spinal cord injury, and may represent a new strategy for treating spinal cord injury.

## Graphical abstract

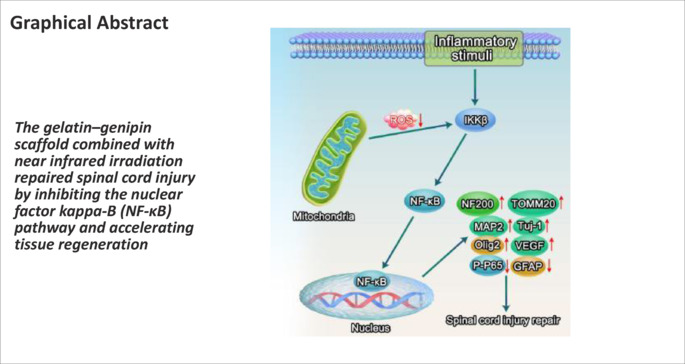

## Introduction

Axonal breaks and neuronal death after spinal cord injury (SCI) result in disabilities (Ma et al., 2022; Yang et al., 2025), and many new cases of SCI are reported annually worldwide (Fan et al., 2022; GBD 2021 Nervous System Disorders Collaborators, 2024). SCI diminishes the lives of patients and incurs substantial medical costs. SCI-induced damage is generally caused by a broad spectrum of secondary injuries following primary mechanical trauma and inflammatory responses (Anjum et al., 2020; Hu et al., 2023; Cheng et al., 2024). First, primary injury induces neuronal death within a short timeframe. Next, secondary injuries such as oxidative stress, apoptosis, neuronal demyelination, inflammation, and scarring can cause additional damage and the functional loss of spinal cord tissue (Hu et al., 2023; Wang and Bai, 2024; Chi et al., 2025). The inflammatory response is a pivotal element in secondary injury and exacerbates tissue damage, thereby hindering neurological recovery. SCI treatments thus aim to suppress inflammation (Su et al., 2025) and promote axonal regeneration in damaged neurons (Gong et al., 2020; Jin et al., 2023b). However, prevalent treatments are relatively ineffective, and the condition has a poor prognosis (Hashimoto et al., 2024; Skinnider et al., 2024; Xu et al., 2024). Recent research in this area has focused on the reconstruction of favorable local microenvironments using biocompatible scaffolds (Yu et al., 2023) that are endowed with desired functions (Luo et al., 2021; Sousa et al., 2025).

Although neuroprotective factors and anti-inflammatory drugs have been used to facilitate axonal growth (Vismara et al., 2020; Xi et al., 2020), their direct delivery is limited in efficacy and fails to promote nerve regeneration (Shan and Wu, 2024). Hydrogels are widely used in biomedical applications (Yan et al., 2024; Zhong et al., 2024; Ghosh et al., 2025). Injectable hydrogels are characterized by good biocompatibility (Arif et al., 2023) and minimal invasiveness, and can be directly administered to the injury site without inducing secondary injury. This renders them ideal biomaterials for the treatment of SCI (Silva et al., 2021; Luo et al., 2022). When acting as a scaffold to fill the lesion cavity, hydrogels facilitate neuronal differentiation. Their porous structures further help to release loading factors and drugs to promote molecular transport within the spinal microenvironment (Wang et al., 2022a, b). Gelatin is non-toxic and used for food preparation (Baumgartner et al., 2020), and therefore constitutes an optimal material. It has been used in various domains, including drug transport and bone tissue engineering (Mushtaq et al., 2022; Andreazza et al., 2023; Chen et al., 2024). Genipin is derived from Gardenia fruit, and is an effective collagen cross-linking agent that has higher biocompatibility and lower cytotoxicity than glutaraldehyde (Huertas-Bello et al., 2023). It can suppress inflammation (Kočí et al., 2020) and act as a crosslinker to form hydrogels, thereby safeguarding their injectability.

Neurotrophic factors can facilitate axonal regeneration after SCI (Zhang et al., 2021). Nerve growth factor (NGF) has multifaceted effects, such as reducing oligodendrocyte death, mitigating demyelination, and offering neuronal protection (Gao et al., 2022; Bao et al., 2023). Total flavonoids from hawthorn leaves (TFHL), which are a group of flavonoids extracted from hawthorn leaves, exhibit anti-inflammatory and antioxidant properties (Zhang et al., 2022).

**Figure 1:**
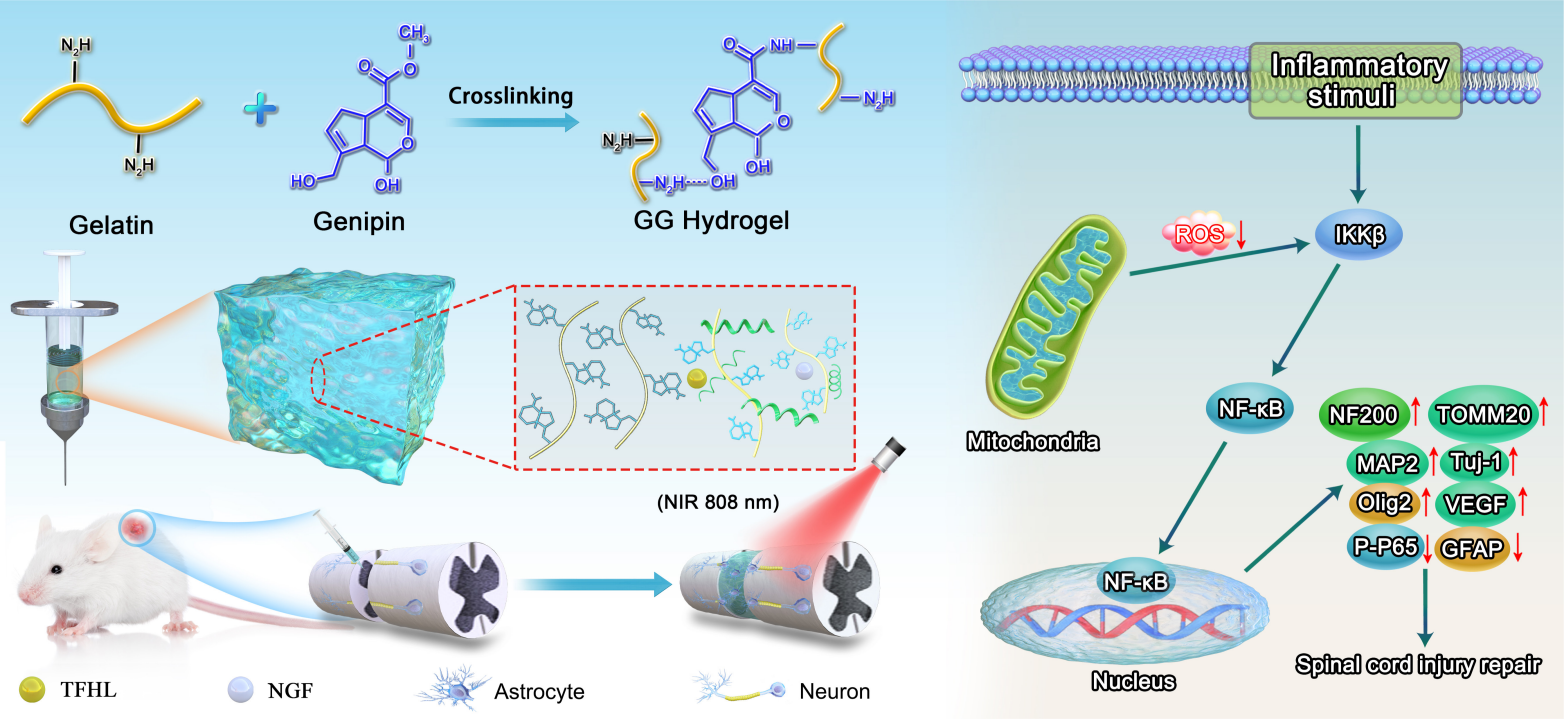
Preparation of a TFHL and NGF dual-loaded GG hydrogel and its NIR irradiation, which enhanced the synergistic effects on spinal cord injury repair. GFAP: Glial fibrillary acidic protein; GG: gelatin-genipin hydrogel; MAP2: microtubule associated protein 2; NF200: neurofilament heavy polypeptide 200; NF-κB: nuclear factor kappa-B; NGF: nerve growth factor; NIR: near infrared; Olig2: oligodendrocyte transcription factor 2; P-P65: phosphorylated P65 protein; ROS: reactive oxygen species; TFHL: total flavonoids from the hawthorn leaves; TOMM20: translocase of outer mitochondrial membrane 20; Tuj-1: class III β-tubulin; VEGF: vascular endothelial growth factor.

On the basis of the aforementioned findings, we developed a hydrogel carrier (GG) by crosslinking gelatin with genipin. We then loaded the GG with NGF and TFHL to form the hydrogel scaffold (GNT) before characterizing its physicochemical properties. The antioxidant, anti-inflammatory, angiogenic, and axonal regeneration behaviors of the GNT were then evaluated, both at the cellular level and in an SCI animal model (**Figure 1**).

## Methods

### Hydrogel preparation and basic characterization

Gelatin powder (Aladdin, Shanghai, China) was dissolved in 10 mL of deionized water to obtain 5%, 10% and 15% (weight/volume [w/v]) gelatin solutions. Genipin powder (Aladdin) was introduced into 1 mL dimethyl sulfoxide (Aladdin) to achieve a concentration of 2% (w/v) genipin solution. Subsequently, different volumes (25, 50, and 75 µL) of genipin solution were separately added to 1 mL of 5%, 10% and 15% of gelatin solution to obtain GG hydrogels, which were defined as 5%, 10%, and 15% GG, respectively. Next, to prepare 10% GN (GG loaded with NGF), 10% GT (GG loaded with TFHL), and 10% GNT (GG loaded with NGF and TFHL) hydrogel scaffolds, NGF or TFHL was separately mixed with 10% GG during hydrogel preparation to achieve a concentration of 200 ng/mL for NGF (Haite Biopharmaceutical, Wuhan, China) or 320 μg/mL for TFHL (Kanglisheng Pharmaceutical, Jincheng, China).

The gelation procedure was observed after mixing the solutions, and was imaged using a camera (Olympus, Tokyo, Japan). The GG hydrogel morphology was then evaluated using a scanning electron microscope (VEGA3, Tescan, Shanghai, China). Samples 0.2 cm in thickness were coated with a 10-nm-thick gold film before the scanning electron microscope observations.

Hydrogel swelling characterization was assessed using the traditional weighing method, as previously described (Feng and Wang, 2023). In brief, the GG hydrogels were freeze-dried for 24 hours. After dehydration, the hydrogels were weighed before being immersed in phosphate-buffered saline (PBS; Aladdin) at 20°C. The soaked hydrogels were then weighed at 0.5, 1, 2, 4, 8, 16, 24, and 48 hours. The swelling ratio was calculated as follows:

Swelling ratio (%) = (*W*_s_ – *W*_d_)/*W*_d_ × 100

Where *W*_s_ represents the mass of the hydrogel in the swelling state and *W*_d_ denotes the mass of the hydrogel at different time points after freeze-drying.

Similarly, the degradation ratio of the GG hydrogels was assessed as follows. Briefly, GG hydrogels were freeze-dried for 1 day. The dehydrated samples were then weighed before they were placed in PBS for 1, 2, 4, 7, 14, 21, and 28 days. The degraded samples were immediately freeze-dried and their degradation ratios were determined as follows:

Degradation ratio (%) = (*W*_o_ – *W*_t_)/*W*_o_ × 100

Where *W*_o_ and *W*_t_ represent the mass of the hydrogel at the beginning and at predetermined time points, respectively.

### Hydrogel physicochemical properties

The rheological properties of the hydrogels were investigated using a rheometer (Bosin Industrial Co. Ltd., Shanghai, China). To understand the storage (G’) and loss (G”) modulus of hydrogels at 10% strain, oscillatory frequency mode measurements were tested in the shear frequency range of 1–100 rad/s at 20°C. For temperature mode measurements, GG hydrogels were tested at 6.28 rad/s and 10% strain in the temperature range of 20–40°C. In particular, the reversibility investigation of 10% GG was implemented at 6.28 rad/s and 20°C with different shear strains (1% or 150%) using a rheometer.

The NGF and TFHL release ratios of the hydrogel scaffolds were assessed as follows. First, the 10% GN hydrogel was incubated in 2 mL of PBS, and the incubated medium was collected at 1, 2, 4, 7, 14, 21, 28, and 35 days. The NGF release ratio was quantified and measured using an NGF enzyme-linked immunosorbent assay (ELISA) kit (Solarbio, Wuhan, China). To evaluate the TFHL release ratio, 10% GT hydrogel was incubated in 2 mL of PBS, and the incubated medium was collected at 1, 2, 4, 7, 14, 21, 28, and 35 days. The TFHL release ratio was measured at 450 mm using a microplate reader (Tecan, Shanghai, China).

To investigate photothermal effects, 10% GG hydrogel was irradiated using a near infrared (NIR) laser (MLL-808, New Industries Optoelectronic Tech, Changchun, China) with a wavelength of 808 nm and a power intensity of 2.5 W/cm^2^. The corresponding images and temperatures were recorded every minute using a thermal imaging camera (FLIR, Wilsonville, OR, USA).

### Biological function assessment at a cellular level

Rat astrocytes were purchased (CP-R306, Punosai Life Technology, Wuhan, China) before being cultured in growth medium comprising high-glucose Dulbecco’s Modified Eagle Medium (DMEM, Gibco, Grand Island, NY, USA) and 15% fetal bovine serum (VWR, Philadelphia, PA, USA). For *in vitro* viability testing, 1 mL of each hydrogel formulation was immersed in 1 mL of DMEM to prepare the hydrogel extracts. The astrocytes were plated at a density of 1 × 10^3^ cells/well in a 96-well plate. After 24 hours of incubation, the cultured medium was replaced with 100 µL of the hydrogel extracts or 100 µL of DMEM (for the control wells). After an additional 24 hours of incubation, 20 µL of Cell Counting Kit-8 reagent (Solarbio) was introduced into each well, and the plate was incubated for a further 4 hours at 37°C in 5% CO_2_. To determine the percentage of live cells, optical density was determined at a wavelength of 450 nm using a microplate reader. In addition, the cells were inoculated into six-well plates (1 × 10^6^ cells/well) before being incubated with 10% GG, 10% GN, or 10% GNT hydrogels for 24 hours. Cytotoxicity was assessed using a live/dead cytotoxicity kit (Solarbio) following the manufacturer’s protocols. Furthermore, the treated cells were trypsinized and suspended in 200 µL of binding buffer. The samples were then treated with 10 mL of Annexin V-fluorescein isothiocyanate and 5 mL of propidium iodide (Beyotime, Shanghai, China) for 10 minutes at room temperature. Next, the samples were adjusted to a total volume of 500 µL by adding binding buffer, and were then quantified using flow cytometry (Beckman Coulter, Brea, CA, USA).

To evaluate their antioxidant and anti-inflammatory abilities, astrocytes were plated in a six-well culture dish at a density of 5 × 10^5^ cells/well. The cells were treated with lipopolysaccharide (LPS, Solarbio) at a concentration of 1 µg/mL for 24 hours, and the medium was then replaced with fresh medium containing 10% GNT hydrogel for an additional 24 hours. Intracellular reactive oxygen species (ROS) levels were assessed using a ROS detection kit (Beyotime) following the manufacturer’s protocols, and the cells were examined under a fluorescence microscope (EVOS M7000, Thermo Fisher Scientific, Waltham, MA, USA). In addition, the cultured medium was collected and inflammatory factor expression levels were analyzed using interleukin (IL)-1β or tumor necrosis factor (TNF)-α ELISA kits (Beyotime) following the manufacturer’s instructions. Inflammatory gene expression levels were also tested using reverse transcription-quantitative polymerase chain reaction (RT-qPCR). Total RNA was isolated from the cell lysis using TRIzol reagent (Beyotime). The RNA was then reverse-transcribed into complementary DNA using a reverse transcription kit (Qihangxing, Beijing, China). The corresponding gene expression levels were detected using a QuantStudio System (Thermo Fisher Scientific). The detailed primer sequences are listed in **Additional Table 1**.

**Additional Table 1:** Primer sequences for reverse transcription-quantitative polymerase chain reaction analysis

Genes	Forward primer (5ʹ-3ʹ)	Reverse primer (5ʹ-3ʹ)
*TNF-α*	ACCATGAGCACGGAAAGCAT	AACTGATGAGAGGGAGCCCA
*IL-1β*	GACTTCACCATGGAACCCGT	GGAGACTGCCCATTCTCGAC
*iNOS*	TCAGGCTTGGGTCTTGTTAGC	CTTGTGGTGAAGGGTGTCGT
*GAPDH*	GCATCTTCTTGTGCAGTGCC	GATGGTGATGGGTTTCCCGT

To investigate the expression levels of nuclear factor kappa-B (NF-κB) signaling pathway-related proteins, western blot analysis was used. In brief, proteins were extracted from the astrocytes using a total protein extraction kit (Solarbio), and the protein contents were detected using a bicinchoninic acid assay (Beyotime). Protein separation was then performed using 10% sodium dodecyl sulfate-polyacrylamide gel electrophoresis. The proteins on the gel were then transferred to polyvinylidene difluoride membranes (Solarbio) using a wet transfer method. Next, membranes were treated with a 5% skim milk solution (Aladdin) in buffer to block non-specific binding. This was followed by an overnight incubation at 4°C with primary antibodies targeting inducible nitric oxide synthase (iNOS; rabbit, 1:1000, Abcam, Shanghai, China, ab178945, RRID: AB_2861417), inhibitor of NF-κB kinase subunit beta (IKKβ; rabbit, 1:1000, Abcam, ab124957, RRID: AB_10975710), nuclear transcription factor (P65; rabbit, 1:1000, CST, Lisle, IL, USA, 8242, RRID: AB_10859369), phosphorylated (P)-P65 (rabbit, 1:1000, CST, 76778, RRID: AB_331284), heat shock 70 kDa protein (HSP70; rabbit, 1:50, Abcam, ab181606, RRID: AB_2910093), and glyceraldehyde 3-phosphate dehydrogenase (GAPDH; rabbit, 1:1000, Abcam, ab181602, RRID: AB_2630358). Next, the membranes were washed three times with Tris-buffered saline with Tween-20 (Aladdin) for 5 minutes, followed by a 1-hour incubation at room temperature with secondary antibody (goat anti-rabbit, 1:500, Servicebio, Wuhan, China, GB25303, RRID: AB_2910224). Finally, the membranes were rinsed again with Tris-buffered saline with Tween-20, detected using a scanner system (Odyssey DLx, LI-COR Biosciences, Lincoln, NE, USA), analyzed using ImageJ (version 1.51, National Institutes of Health, Bethesda, MD, USA) (Schneider et al., 2012), and normalized against the internal reference.

### *In vivo* animal experiments

Sprague–Dawley rats (female, 4 weeks old, 150–180 g) were obtained from the Laboratory Animal Center at Guangxi Medical University (license No. SYXK [Gui] 2020-0004). All animal experiments were approved by the ethical review board of Guangxi Medical University (approval No. 202308119). The experiments were designed and reported according to the Animal Research: Reporting of *In Vivo* Experiments (ARRIVE) guidelines (Percie du Sert et al., 2020).

Fifty female rats were randomly assigned to five groups: SCI, 10% GG, 10% GN, 10% GNT, and 10% GNT + NIR (*n* = 10 rats per group). The selection of only female rats for the SCI experiments was mainly due to the fact that they have fewer postoperative complications, better neurological function recovery, a smaller inflammatory response, and more stable experimental results (Datto et al., 2015; Hellenbrand et al., 2024).

The rats were anesthetized using sodium pentobarbital (0.1 mL/100 g, Jiehui Biotechnology, Beijing, China) via intravenous administration. The T9–T11 vertebral arch was excised, and the spinal cord was sectioned using ophthalmic scissors to create a gap of approximately 1 mm. Thereafter, approximately 15 µL of hydrogel solution was administered intraperitoneally into all groups except for the SCI group, who received an equivalent volume of physiological saline solution. After the operation, manual assistance with urination was provided to the rats twice daily until they regained the ability to urinate independently. For the 10% GNT + NIR group, NIR irradiation was performed daily for 10 minutes using an NIR laser (808 nm, 2.5 W/cm^2^) for 4 weeks. At 28 days after treatment, rats were euthanized. Following pentobarbital anesthesia, the left ventricle was rapidly perfused with 200 mL of normal saline followed by 100 mL of 4% paraformaldehyde (Biosharp, Hefei, China) for fixation.

Major organs, including the heart, liver, spleen, lung, kidney, and whole blood from the rats were extracted on day 28; these were subjected to hematoxylin and eosin (H&E) staining and biochemical analysis. The blood samples were then used to evaluate routine blood (veterinary fully automatic blood cell analyzer, Mindray, Shenzhen, China) and blood biochemical indicators as well as liver and kidney functions (fully automatic biochemical analyzer, Thermo Fisher Scientific).

Spinal cord tissue samples were fixed with paraformaldehyde before being dehydrated in different concentrations of ethanol (Aladdin). The dehydrated tissue was placed into xylene (Aladdin) before being embedded in wax blocks. The wax blocks were cut into thin slices and placed onto slides using a sectioning machine (DTK-ZERO 1N, Dosaka, Kyoto, Japan). They were then stained using an H&E Staining Kit (G1076, Servicebio) and observed under a microscope (Olympus). In addition, the spine cord tissue samples underwent immunofluorescent staining with the following primary antibodies: P65 (rabbit, 1:500, CST, 8242, RRID: AB_10859369), neurofilament protein 200 (NF200; mouse, 1:500, SA, St. Louis, MO, USA, N0142, RRID: AB_477257), oligodendrocyte transcription factor 2 (OLIG2; rabbit, 1:500, Abcam, ab109186, RRID: AB_10861310), beta-3 tubulin (TUJ-1; rabbit, 1:1000, Abcam, ab18207, RRID: AB_444319), glial fibrillary acidic protein (GFAP; rabbit, 1:5000, Abcam, ab7260, RRID: AB_305808), translocase of outer mitochondrial membrane 20 (TOMM20; rabbit, 1:500, Abcam, ab186734, RRID: AB_2716623), microtubule-associated protein 2 (MAP2; rabbit, 1:500, Abcam, ab183830, RRID: AB_776174), or vascular endothelial growth factor (VEGF; rabbit, 1:100, Abcam, ab2349, RRID: AB_302998) at 4°C overnight. The samples were then incubated with secondary goat anti-rabbit (Alexa Fluor 488, goat anti-rabbit immunoglobulin [Ig]G, 1:100, Servicebio, GB25303, RRID: AB_2910224) or goat anti-mouse (Alexa Fluor 488, goat anti-mouse IgG, 1:100, Servicebio, GB25301, RRID: AB_2904018) antibody for 12 hours at 4°C. After washing again with PBS, the sections were analyzed using a confocal microscope (ZEISS, Oberkochen, Germany).

Finally, the Basso–Beattie–Bresnahan (BBB) locomotion scale was used to assess the locomotive recovery of rats at 0, 7, 14, and 28 days. The BBB scale was scored from 0–21 points. Gait analysis was also performed using the CatWalk system (VisuGait, Shanghai, China) at 28 days post-injury. The results of the regularity index and base of support were obtained from the system, and were analyzed by three independent evaluators.

### Statistical analysis

Quantitative analyses are presented as the mean ± standard deviation. To determine significance, one-way analysis of variance followed by Tukey’s *post hoc* test was used to conduct comparisons across multiple groups. All statistical analyses were performed using IBM SPSS Statistics version 26.0 (IBM, Armonk, NY, USA). *P* < 0.05 was established as the criterion for significance.

## Results

### Preparation and physicochemical properties of hydrogels

**Figure 2:**
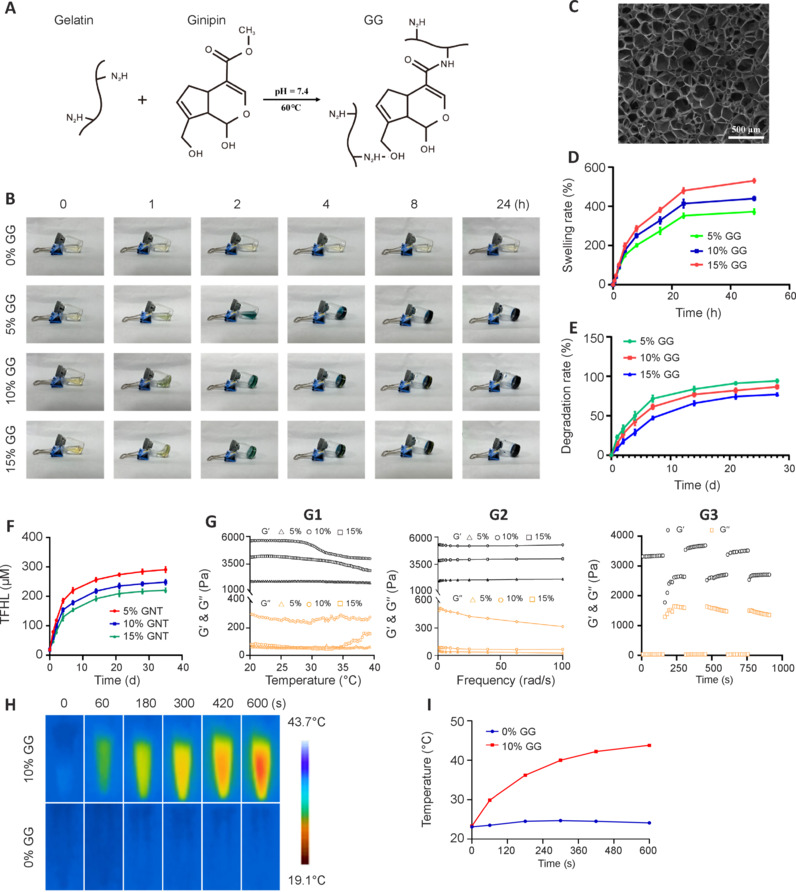
Preparation and physicochemical characterization of GNT hydrogels. (A) Chemical synthesis diagram of GG. (B) Gelation procedures of different GG. (C) SEM image of 10% GG. The hydrogels exhibited a well-developed porous architecture characterized by interconnected pores that varied in size. Scale bar: 500 µm. (D) Swelling rates of different GG. (E) Degradation rates of different GG. (F) TFHL release rates of different GNT. (G) Rheological behaviors of different GG in three modes: temperature mode (6.28 rad/s and 10% strain in a temperature range of 20–40°C) (G1), frequency mode (shear frequency range of 1–100 rad/s at 20°C) (G2), and reversibility (6.28 rad/s and 20°C) with different shear strains (1% or 150%) (G3). (H) Photothermal images of 0% GG and 10% GG under NIR laser (808 nm, 2.5 W/cm^2^). 10% GG had a significant photothermal effect, with corresponding temperature changes over time (I). G′: Elastic modulus; G′′: viscous modulus; GG: gelatin-genipin hydrogel; GN: NGF-loaded GG; GNT: NGF and TFHL dual-loaded GG; NGF: nerve growth factor; NIR: near infrared; SEM: scanning electron microscope; TFHL: total flavonoids from the hawthorn leaves.

Hydrogels were prepared by mixing the genipin and gelatin solutions. The ester groups of genipin reacted with the amino groups of gelatin, and the hydroxyl groups of genipin formed hydrogen bonds with the amino groups of gelatin; these both contributed to the formation of the crosslinking network in the hydrogels (**Figure 2A**). During the gelation process, 0% GG (without genipin mixing) was unable to form a hydrogel after 24 hours. Conversely, after mixing with the genipin solution, hydrogels were fabricated in approximately 2 hours for 5%, 10%, and 15% GG, and their color became darker over time (**Figure 2B**). Furthermore, 10% GG hydrogels exhibited excellent injectability with a syringe (**Additional Figure 1A**); they also displayed some adhesive ability (**Additional Figure 1B**). Importantly, a clear interpenetrated network was observed in 10% GG, with pore sizes ranging during 20–150 µm (**Figure 2C**).

As displayed in **Figure 2D**, hydrogels swelled gradually over time. After 48 hours, the swelling rates were 387%, 450%, and 540% for 15%, 10%, and 5% GG, respectively (**Figure 2D**). By contrast, the hydrogels exhibited rapid degradation during the initial week, and the degradation rate markedly decelerated in the following weeks. After 4 weeks, the complete degradation of hydrogels was not achieved, with approximately 10% of hydrogels remaining undegraded (**Figure 2E**). Increasing the concentration of genipin—equal to increasing the crosslinking degree of hydrogels—always contributed to lower swelling and degradation rates.

Spectrometry was used to assess the prolonged release profile of TFHL from the GG hydrogels. As shown in **Figure 2F**, TFHL release was higher during the initial week, followed by a subsequent deceleration in the release rate. In addition, 5% GG was observed to possess a higher TFHL release than 10% and 15% GG. The standard curve of TFHL is illustrated in **Additional Figure 1C**. ELISA was used to assess the sustained release of NGF. Compared with the NGF standard curve (**Additional Figure 1D**), GG hydrogels exhibited slow drug release until 36 hours (**Additional Figure 1E**).

The rheological properties of the hydrogels were investigated using three modes. Although the storage (G’) modulus of 10% GG and 15% GG varied with temperature differences, the loss (G”) modulus of all hydrogels maintained the same trend within a temperature range of 20–40 °C (**Figure 2G1**). However, the G’ was higher than the G” in all hydrogels, indicating the elastic properties of the hydrogels. The G’ of the hydrogels remained stable and exceeded G” across an angular frequency of 1–100 rad/s. This finding indicates that, within this frequency range, the hydrogels preserve their initial network configurations (**Figure 2G2**). Notably, variations of G’ and G” occurred in 10% GG with different shear strains (1% and 150%), which indicates the self-healing properties of 10% GG. Both G’ and G” remained stable after four cycles, further suggesting that no new permanent deformation occurred in 10% GG (**Figure 2G3**).

Next, the photothermal response of 10% GG was examined under irradiation with an NIR laser at a wavelength of 808 nm (Lei et al., 2025). The temperature increased versus the irradiation time in 10% GG, but not in 0% GG (**Figure 2H**). Statistical analysis revealed that the temperature increased from 23.3–43.8°C in 10% GG after 10 minutes of irradiation but remained stable in 0% GG (**Figure 2I**).

### Biological functions of hydrogel scaffold at a cellular level

**Figure 3:**
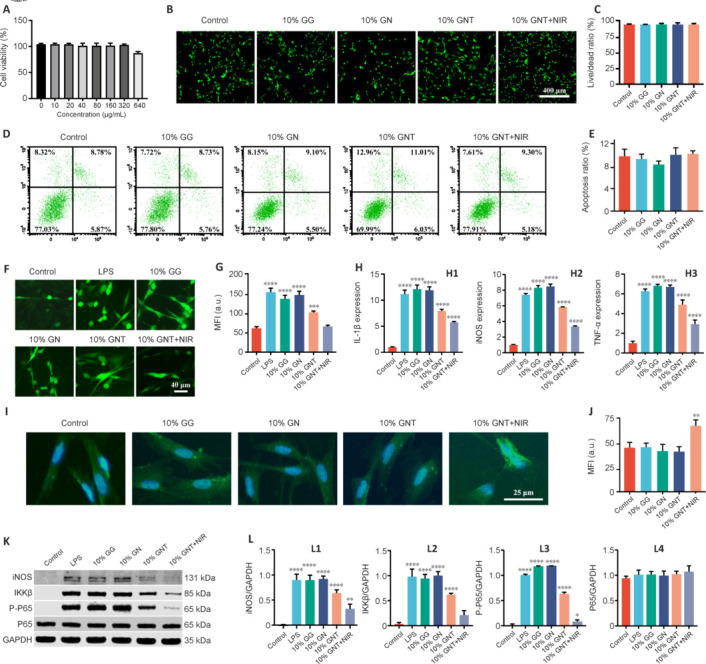
Biological function of 10% GNT at a cellular level. (A) Cell viability of 10% GNT with different concentrations of TFHL using CCK-8. (B, C) Live/dead staining images of different hydrogel-treated cells (B), and the corresponding quantitative results (C). Scale bar in B: 400 µm. (D) Cell apoptosis conditions of treated cells using flow cytometry, and the corresponding apoptosis ratios (E). (F, G) Intracellular ROS levels of treated cells using fluorescent staining (F), and the corresponding MFI (G). Scale bar in F: 40 µm. (H) Inflammation-related gene expression levels of treated cells using RT-qPCR: IL-1β (H1), iNOS (H2), and TNF-α (H3). (I, J) Immunofluorescent images of HSP70 expression in treated cells and the corresponding MFI of HSP70. Scale bar in I: 25 µm. (K) WB images of iNOS, IKKβ, P-P65, P65, and GAPDH expression levels in spinal cord sections from treated rats, and the corresponding quantified results (L): iNOS/GAPDH ratio (L1), IKKβ/GAPDH ratio (L2), P-P65/GAPDH ratio (L3), and P65/GAPDH ratio (L4). Data are expressed as the mean ± standard deviation (*n* = 3 independent experiments). The groups were: cells without treatment (control group); LPS-induced cells (LPS group); and LPS-induced cells co-incubated with 10% GG (10% GG), 10% GN (10% GN), 10% GNT (10% GNT), and 10% GNT combined with NIR irradiation (10% GNT + NIR). *P < 0.05, **P < 0.01, ***P < 0.001, ****P < 0.0001 (one-way analysis of variance followed by Tukey’s *post hoc* test). a.u.: Arbitrary unit; CCK-8: cell counting kit-8; GAPDH: glyceraldehyde-3-phosphate dehydrogenase; GG: gelatin-genipin hydrogel; GN: NGF loaded GG; GNT: NGF and TFHL dual-loaded GG; HSP70: heat shock protein 70; IKKβ: inhibitor of kappa B kinase beta; IL-1β: interleukin-1 beta; iNOS: inducible nitric oxide synthase; LPS: lipopolysaccharide; MFI: mean fluorescent intensity; NGF: nerve growth factor; NIR: near infrared; P-P65: phosphorylated P65; ROS: reactive oxygen species; RT-qPCR: reverse transcription-quantitative polymerase chain reaction; SCI: spinal cord injury; TFHL: total flavonoids from the hawthorn leaves; TNF-α: tumor necrosis factor-alpha; WB: western blotting.

The cell viability of 10% GNT with different TFHL concentrations was assessed using the Cell Counting Kit-8 method. Cell viability was nearly 100% with TFHL concentrations below 320 µg/mL (**Figure 3A**), demonstrating the minimal cytotoxic effects of GNT. Thus, in all further experiments, 320 µg/mL of TFHL was applied in 10% GNT. Furthermore, many living cells and just a few dead cells were observed after a 3-day incubation (**Figure 3B**), with a live/dead ratio of approximately 100% (**Figure 3C**). This finding indicates the excellent biocompatibility of all groups. The cytotoxic effects of the hydrogels were then assessed using flow cytometry. There were no marked differences in the apoptosis ratio among all groups, with an apoptosis ratio below 10% (**Figure 3D** and **E**). Together, these findings indicate the biocompatibility of our hydrogels.

The antioxidant and anti-inflammatory capacities of hydrogels were evaluated *in vitro*. As shown in **Figure 3F**, intracellular ROS levels were relatively low in normal astrocytes (control group), with very little green fluorescence observed. There was a notable increase in green fluorescence (i.e., ROS levels) in LPS-stimulated astrocytes (LPS group), whereas a reduction in green fluorescence was detected in the 10% GNT and 10% GNT + NIR groups. However, 10% GG and 10% GN did not effectively inhibit ROS generation. Statistical analysis indicated that the 10% GNT hydrogel was indeed effective at ROS inhibition, with a clear reduction in mean fluorescent intensity compared with the LPS group, and the ROS scavenging ability was significantly enhanced in the 10% GNT + NIR group (**Figure 3G**).

The anti-inflammatory capacities were initially evaluated using ELISA to test the expression levels of inflammatory factors in the supernatant of treated cells. There was substantially higher IL-1β and TNF-α expression in the LPS group than in the control group (*P* < 0.0001). Conversely, these cytokine levels were markedly reduced in both the 10% GNT and 10% GNT + NIR groups (*P* < 0.0001). No significant variations were observed in IL-1β or TNF-α levels across the LPS, 10% GG, and 10% GN groups (**Additional Figure 2A** and **B**). We also quantified the expression levels of inflammation-related genes in treated astrocytes using RT-qPCR. As illustrated in **Figure 3H**, the gene expression levels of IL-1β, iNOS, and TNF-α were highest in the LPS group and lowest in the control group. Notably, the 10% GG and 10% GN groups did not have reduced gene expression levels; their levels were close to those of the LPS group. Nevertheless, both 10% GNT and 10% GNT + NIR effectively lowered the gene expression levels of IL-1β, iNOS, and TNF-α, and 10% GNT + NIR exhibited the most efficient reduction in inflammatory gene expression.

The HSP70 expression levels in treated cells were evaluated using immunofluorescent staining. HSP70 expression (green fluorescence) was relatively low in all groups except the 10% GNT + NIR group, which had high HSP70 expression (**Figure 3I** and **J**). This result indicates a good photothermal therapeutic effect of 10% GNT + NIR.

Finally, the expression levels of NF-κB signaling pathway-related proteins were assessed in treated astrocytes using western blot analysis. There was a significant upregulation of iNOS, IKKβ, and P-P65 in the LPS group compared with the control group (**Figure 3K** and **L**). This result suggests that LPS triggers the NF-κB pathway in astrocytes. By contrast, the 10% GNT group exhibited substantially lower expression of these proteins compared with the LPS group. A comparison between the 10% GNT and 10% GNT + NIR groups revealed significant differences in their iNOS, IKKβ, and P-P65 expression, indicating that NIR irradiation-induced exogenous hyperthermia strengthens the drug release and anti-inflammatory effects of 10% GNT. However, there were no significant differences in iNOS, IKKβ, or P-P65 expression among the LPS, 10% GG, and 10% GN groups, and there were no differences in P65 expression among all groups. Collectively, these findings suggest that 10% GNT can suppress the expression levels of NF-κB signaling pathway-related proteins.

### *In vivo* therapeutic effects of 10% hydrogel scaffold in the spinal cord injury rat model

**Figure 4:**
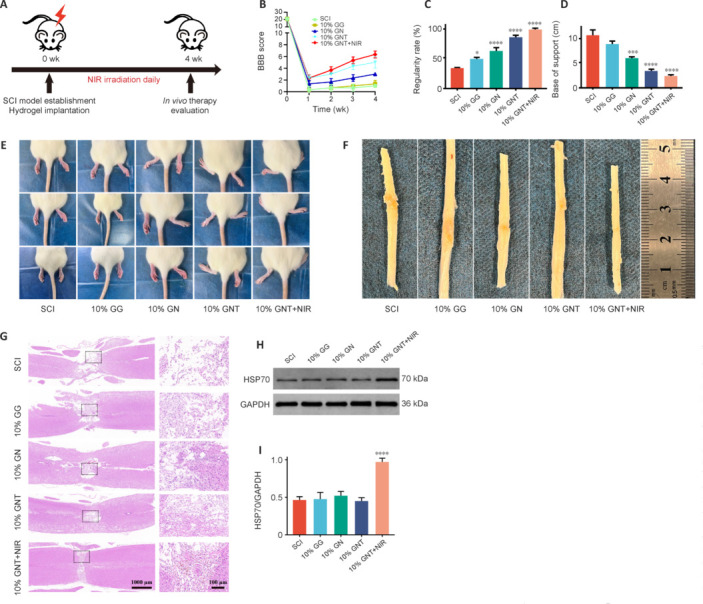
*In vivo* evaluation of 10% GNT in the SCI rat model. (A) Schedule of the *in vivo* animal experiments. (B) BBB scores of treated rats using gait analysis. (C) Regularity rates of treated rats using gait analysis. (D) Bases of support of treated rats using gait analysis. (E) Macroscopic observations of the backs of treated rats. (F) Macroscopic observations of the spinal cords of treated rats. (G) H&E-stained images of spinal cord sections from treated rats. (H) WB images of HSP70 expression in spinal cord sections from treated rats, and the corresponding HSP70/GAPDH ratios (I). Scale bars: 1000 µm and 100 µm. The groups were: SCI rats with PBS injection (SCI group), 10% GG injection (10% GG), 10% GN injection (10% GN), 10% GNT injection (10% GNT), and 10% GNT injection combined with NIR irradiation (10% GNT + NIR). Data are expressed as the mean ± standard deviation (*n* = 6 independent experiments). *P < 0.05, ***P < 0.001, ****P < 0.0001 (one-way analysis of variance followed by Tukey’s *post hoc* test). BBB: Basso-Beattie-Bresnahan; GAPDH: glyceraldehyde-3-phosphate dehydrogenase; GG: gelatin-genipin hydrogel; GN: NGF-loaded GG; GNT: NGF and TFHL dual-loaded GG; HSP70: heat shock protein 70; H&E: hematoxylin and eosin; NGF: nerve growth factor; NIR: near infrared; SCI: spinal cord injury; TFHL: total flavonoids from the hawthorn leaves; WB: western blotting.

The schedule of the *in vivo* animal experiments is listed in **Figure 4A**. After SCI model establishment, the rats were implanted with hydrogels before being exposed to NIR irradiation daily for 4 weeks. To evaluate *in vivo* biosafety, blood indicators and histological staining of the main organs of treated rats were investigated on day 28 after therapy. After 4 weeks of treatment, blood indicators including white blood cells, red blood cells, platelets, hemoglobin, total bilirubin, alanine aminotransferase, aspartate aminotransferase, albumin, uric acid, urea, creatinine, and blood urea nitrogen showed no substantial variations in the 10% GG, 10% GN, 10% GNT, and 10% GNT + NIR groups compared with the SCI group (**Additional Figure 3**). This finding suggests that the 10% GNT group did not have an increased risk of acute infection, hemolytic anemia, or liver and kidney dysfunction. In addition, H&E-stained images of the heart, liver, spleen, lung, and kidney revealed no signs of pathological changes (**Additional Figure 4**), indicating that hydrogel implantation does not noticeably impair vital organs. Together, these results highlight the excellent biocompatibility of our hydrogels *in vivo*.

The locomotor function of treated rats was also analyzed using data from their post-operative movements, including gait stability analysis and BBB scores. The hind limbs of all rats were completely paralyzed after the operation, but the movement of these limbs gradually increased. Compared with the 10% GG and SCI groups, the 10% GNT and 10% GNT + NIR groups had higher BBB scores. The motor function recovery speed was in the order of 10% GNT + NIR > 10% GNT > 10% GN > 10% GG and SCI (**Figure 4B**). Furthermore, the CatWalk assessment results indicated that 10% GNT hydrogel improved rat motor function compared with the other treatments (**Additional Figure 5**). In the statistical analysis, the 10% GNT + NIR group had the highest regularity index and lowest base of support, whereas the SCI group had the lowest regularity index and the highest base of support (**Figure 4C** and **D**). These findings further indicate that 10% GNT hydrogels significantly improve hindlimb function and further SCI recovery.

**Figure 5:**
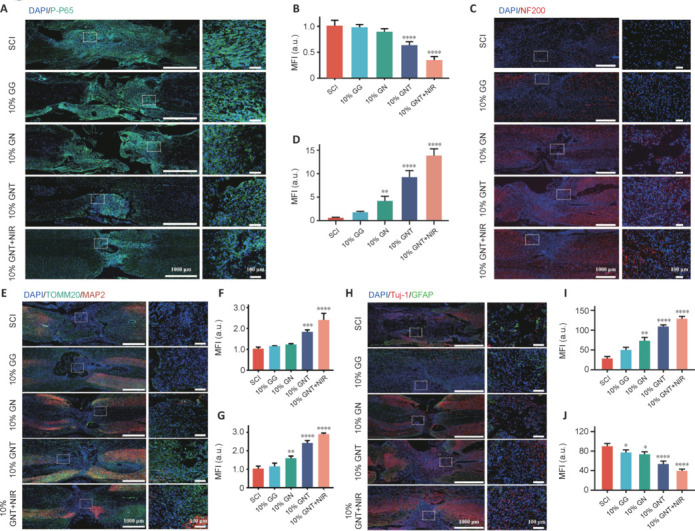
NIR and 10% GNT synergistically enhance the NF-κB pathway in SCI rats. (A, B) Immunofluorescent images of P-P65 in the spinal cords of treated rats (A), and the corresponding MFI of P-P65 expression (B). (C, D) Immunofluorescent images of NF200 in the spinal cords of treated rats (C) and the corresponding MFI of NF200 (D). (E) Co-immunofluorescent images of TOMM20 and MAP2 in the spinal cords of treated rats, and the corresponding MFI of TOMM20 (F) and MAP2 (G). (H) Co-immunofluorescent images of TUJ-1 and GFAP expression in the spinal cords of treated rats, and the corresponding MFI of TUJ-1 (I) and GFAP (J) expression. The groups were: SCI rats with PBS injection (SCI group), 10% GG injection (10% GG), 10% GN injection (10% GN), 10% GNT injection (10% GNT), and 10% GNT injection combined with NIR irradiation (10% GNT + NIR). Scale bars: 1000 µm and 100 µm in A, C, E and H. Data are expressed as the mean ± standard deviation (*n* = 6 independent experiments). *P < 0.05, **P < 0.01, ***P < 0.001, ****P < 0.0001 (one-way analysis of variance followed by Tukey’s *post hoc* test). a.u.: Arbitrary unit; DAPI: 4′,6-diamidino-2-phenylindole; GFAP: glial fibrillary acidic protein; GG: gelatin-genipin hydrogel; GN: NGF loaded GG; GNT: NGF and TFHL dual-loaded GG; MFI: mean fluorescent intensity; NF200: neurofilament heavy polypeptide 200; NGF: nerve growth factor; NIR: near infrared; P-P65: phosphorylated P65 protein; SCI: spinal cord injury; TFHL: total flavonoids from the hawthorn leaves; TOMM20: translocase of outer mitochondrial membrane 20; Tuj-1: class III β-tubulin.

In the macroscopic observation of the backs of rats, no significant differences were observed among all groups (**Figure 4E**). After removing the spinal cord, clear scar hyperplasia was observed in the SCI, 10% GG, and 10% GN groups. However, we observed a reduction in scar hyperplasia for both the 10% GNT and 10% GNT + NIR groups compared with the SCI group (**Figure 4F**). Similarly, in H&E-stained tissue, the SCI and 10% GG groups had clear, broad lesion areas, whereas the 10% GNT and 10% GNT + NIR groups had notably smaller lesion areas (**Figure 4G**). These findings suggest that 10% GNT and 10% GNT + NIR effectively inhibit the formation of cavities and scarring in SCI rats. Additionally, HSP70 was more highly expressed in the 10% GNT + NIR group than in the other groups (**Figure 4H** and **I**).

We next investigated the expression levels of inflammation-related proteins using immunofluorescent staining of the spinal cord. **Figure 5A** shows that P-P65 expression was lower in the 10% GNT and 10% GNT + NIR groups than in the SCI and 10% GG groups. Moreover, there was slightly lower green fluorescence in the 10% GN group than in the SCI group. Among all groups, 10% GNT + NIR was the most effective at lowering P-P65 expression and inhibiting inflammatory reactions (**Figure 5B**). We also analyzed NF200 expression. The 10% GNT and 10% GNT + NIR groups promoted nerve growth, with the strongest NF200 signal at the boundary of the injury sites; these levels markedly surpassed those observed in the 10% GG group. Almost no NF200 intensity was detected in the SCI group (**Figure 5C**). Together, these findings indicate that hydrogel treatments effectively promote nerve fiber growth in the area of spinal cord lesions, with a mean fluorescent intensity order of 10% GNT + NIR > 10% GNT > 10% GN > 10% GG > SCI (**Figure 5D**). The expression of mitochondrial function-related proteins was also assessed using immunofluorescent staining. There was a substantial reduction in the fluorescent intensity of both TOMM20 and MAP2 following SCI. After intervention, their expression was highest in the 10% GNT + NIR group compared with all other groups (**Figure 5E–G**), indicating that 10% GNT + NIR treatment is the most efficient for improving mitochondrial function and promoting neuronal cell maturation.

Finally, immunofluorescent TUJ-1 and GFAP staining was performed to observe axonal regeneration and scar proliferation at the injury site. The fluorescent intensity of TUJ-1 was higher in the 10% GNT and 10% GNT + NIR groups than in the SCI, 10% GG, and 10% GN groups. Conversely, GFAP expression was significantly higher in the SCI and 10% GG groups than in the other groups (**Figure 5H**). Glial scar formation presented a strong barrier to nerve regeneration and functional restoration, limiting astrocyte proliferation. Notably, the 10% GNT + NIR group had the highest TUJ-1 expression and the lowest GFAP expression (**Figure 5I** and **J**). In addition, OLIG2-stained images exhibited more oligodendrocyte regeneration and remyelination in the 10% GNT and 10% GNT + NIR groups than in the other groups (**Additional Figure 6A** and **B**). In the VEGF-stained images, more red fluorescence was observed in the 10% GNT and 10% GNT + NIR groups than in the SCI, 10% GG, and 10% GN groups (**Additional Figure 6C** and **D**). This result suggests that the 10% GNT and 10% GNT + NIR groups may promote the formation of new blood vessels, which are vital for spinal cord repair. Together, these findings indicate that 10% GNT + NIR treatment may most efficiently reduce glial scar formation and enhance axonal growth and the development of new blood vessels at the injury site.

## Discussion

The development of an effective SCI treatment strategy is challenging because of the complex pathophysiological processes that are involved, including inflammation, oxidative stress, and secondary tissue damage. In the present study, we designed and evaluated a TFHL and NGF dual-loaded hydrogel scaffold (GNT) based on gelatin and genipin. We then combined this scaffold with NIR irradiation for SCI repair.

Our physicochemical results indicate that 10% GG exhibits excellent drug carrier behaviors, including injectability, drug-controlled release, and photothermal effects; these collectively contribute to enhancing SCI repair. The porous structure of GG facilitated cellular infiltration and substance exchange. Additionally, because the G’ of the hydrogel scaffolds closely matched that of spinal cord tissue, the hydrogels adapted to normal tissue without causing secondary damage from compression (Ramo et al., 2018; Jin et al., 2023a). The G’ of all hydrogels fell within 2–5 kPa, which is closely aligned with the stiffness of central nervous system tissues, which range from 0.1–16 kPa. This similarity in mechanical properties represents an optimal setting for neuronal differentiation (Li et al., 2022). The high adhesive properties of GG also contribute to improved SCI repair (Liang et al., 2021). Furthermore, the sustained release of NGF and TFHL from GNT provided a continuous supply of neurotrophic and anti-inflammatory factors, which are crucial for promoting nerve regeneration and reducing inflammation. A rapid initial drug release followed by a sustained release reportedly facilitates SCI repair (Luo et al., 2021). Importantly, the photothermal effects of GG further enhanced drug release and therapeutic efficacy in the present study, as demonstrated by the high HSP70 expression. High HSP70 expression also inhibited NF-κB signaling pathway activation, suppressed neuroinflammation, and promoted neuronal regeneration. These findings suggest that GG has strong photothermal effects, which may promote its therapeutic effects (Xue et al., 2021; Yeo et al., 2022; Hu et al., 2024).

At the cellular level, 10% GG, 10% GN, and 10% GNT displayed good biocompatibility, which is a critical property in SCI repair (Gao et al., 2023). ROS also plays a crucial role in cell signaling pathways (Joorabloo and Liu, 2024; Tang et al., 2024) such as the NF-κB signaling pathway. In the context of SCI, the inflammatory microenvironment is crucially shaped by IL-6, IL-1β, and TNF-α (Li et al., 2023). Moreover, the NF-κB signaling pathway is critical for cellular communication, as well as a myriad of physiological processes, including immune reactions, inflammatory responses, and the regulation of cell proliferation and apoptosis (Xin et al., 2023). Reprogramming the inflammatory microenvironment of the spinal cord promotes neuronal growth (Wang et al., 2021). Thus, 10% GNT may effectively inhibit the inflammatory response in LPS-stimulated astrocytes by decreasing intracellular ROS levels, lowering inflammatory factor expression, inhibiting the NF-κB signaling pathway, and promoting repair factor expression. These findings highlight the potential of 10% GNT for modulating the inflammatory microenvironment in SCI. Notably, the aforementioned conditions were enhanced by NIR irradiation, similar to earlier reports. For example, photothermal therapy has been reported to enhance overall therapeutic effectiveness (Liang et al., 2024).

Preservation of the structural and functional aspects of mitochondria also plays a pivotal role in neuronal survival. Inhibiting inflammation enhances mitochondrial performance and promotes axonal repair (Hashimoto et al., 2024). TOMM20 is an important protein located in the outer mitochondrial membrane, and MAP2 is predominantly expressed in fully developed neurons. OLIG2 is a marker of both early and mature oligodendrocytes and is important for many different physiological activities (He et al., 2022). Notably, glial scar formation presents a strong barrier to nerve regeneration and functional restoration (Milich et al., 2021; Liu et al., 2024) and especially limits astrocyte proliferation. Moreover, the formation of new blood vessels is essential for spinal cord repair. In our SCI rat model, histological and functional assessments suggested that the hydrogels have good therapeutic efficacy. Histological analysis revealed reduced lesion areas, decreased glial scar formation, increased blood vessels, and increased nerve fiber regeneration in rats treated with 10% GNT + NIR. Collectively, these results indicate that 10% GNT not only mitigates secondary damage but also promotes tissue repair and functional recovery, and its effects are enhanced by NIR irradiation.

Despite our promising results, the present study has several limitations. It remains unclear precisely how the GNT hydrogel regulates the NF-κB signaling pathway, particularly in terms of the molecular mechanisms; these require further investigation. Additionally, although the hydrogel exhibited excellent biocompatibility, the long-term effects of its implantation *in vivo* need evaluation in larger animal models, and eventually in clinical trials. Finally, the translation of our approach to SCI treatments in human patients will require the careful consideration of 1) injury types, and 2) the potential risks associated with hydrogel injection.

In summary, a TFHL and NGF dual-loaded hydrogel scaffold based on gelatin and genipin was developed as a drug delivery system for SCI repair. Our results indicate that 10% GG exhibits optimal gel-forming properties, injectability, rheological characteristics, and photothermal effects. At a cellular level, 10% GNT combined with NIR irradiation led to the controlled release of TFHL and NGF and had robust antioxidant and anti-inflammatory capabilities. These effects occurred via the reduction of intracellular ROS levels, suppression of inflammatory factor expression, inhibition of the NF-κB signaling pathway, and upregulation of tissue repair factor expression, thereby enhancing SCI repair. Notably, 10% GNT + NIR treatment amplified the suppression of inflammatory responses, as well as the promotion of angiogenesis, remyelination, and nerve fiber regeneration. These effects ultimately reprogrammed the spinal cord microenvironment and accelerated SCI repair. Overall, our findings suggest a novel strategy for improved SCI therapy, and have potential applications for treating other inflammation-related diseases.

## Additional files:

***Additional Figure 1:*** *Characterization of hydrogels.*

Additional Figure 1 Characterization of GN hydrogels.
(A) Injectability of 10% GG. (B) Adhesive ability of 10% GG. (C) Standard curve of TFHL. (D) Standard curve of NGF. (E) NGF release rates of 5%, 10%, and 15% GN. Data are expressed as the mean ± standard deviation (*n* = 3 independent experiments). GG: Gelatin-genipin hydrogel; GN: NGF loaded GG; NGF: nerve growth factor; TFHL: total flavonoids from the hawthorn leaves.

***Additional Figure 2:*** *Inflammatory factor expression levels in the supernatant of astrocytes using ELISA.*

Additional Figure 2 Inflammatory factor expression levels in the supernatant of astrocytes using ELISA.
(A) IL-1β expression levels in the supernatant of treated astrocytes using ELISA. (B) TNF-α expression levels in the supernatant of treated astrocytes using ELISA. Data are expressed as the mean ± standard deviation (*n* = 3 independent experiments). *P < 0.05, **P < 0.01, ***P < 0.001, ****P < 0.0001 (one-way analysis of variance followed by Tukey's *post hoc* test). The groups were: cells without treatment (control group); LPS-induced cells (LPS group); and LPS-induced cells co-incubated with 10% GG (10% GG), 10% GN (10% GN), 10% GNT (10% GNT), and 10% GNT combined with NIR irradiation (10% GNT + NIR). ELISA: Enzyme-linked immunosorbent assay; GG: gelatin-genipin hydrogel; GN: NGF loaded GG; GNT: NGF and TFHL dual-loaded GG; IL-1β: interleukin-1 beta; LPS: lipopolysaccharide; NGF: nerve growth factor; NIR: near infrared; TFHL: total flavonoids from the hawthorn leaves; TNF-α: tumor necrosis factor-alpha.

***Additional Figure 3:*** *Blood indictors of SCI rat models treated with 10% GNT.*

Additional Figure 3 Blood indicators in SCI rat models treated with 10% GNT.
The groups were: SCI rats with PBS injection (SCI group), 10% GG injection (10% GG), 10% GN injection (10% GN), 10% GNT injection (10% GNT), and 10% GNT injection combined with NIR irradiation (10% GNT + NIR). Data are expressed as the mean ± standard deviation (*n* = 6 independent experiments). ALB: Albumin; ALT: alanine aminotransferase; AST: aspartate aminotransferase; BUN: blood urea nitrogen; CREA: creatinine; GG: gelatin-genipin hydrogel; GN: NGF loaded GG; GNT: NGF and TFHL dual-loaded GG; HGB: hemoglobin; NGF: nerve growth factor; NIR: near infrared; PLT: platelet; RBC: red blood cell; SCI: spinal cord injury; TBIL: total bilirubin; TFHL: total flavonoids from the hawthorn leaves; UA: uric acid; WBC: white blood cell.

***Additional Figure 4:*** *H&E staining images of major organs (heart, liver, spleen, lung, and kidney) of SCI rat models treated with 10% GNT.*

Additional Figure 4 H&E staining images of major organs (heart, liver, spleen, lung, and kidney) of SCI rat models treated with 10% GNT.
The groups were: SCI rats with PBS injection (SCI group), 10% GG injection (10% GG), 10% GN injection (10% GN), 10% GNT injection (10% GNT), and 10% GNT injection combined with NIR irradiation (10% GNT + NIR). Normal organ structures were present in all five groups of rats, no inflammatory cell infiltration was observed, and there were no clear pathological changes among the groups. Scale bar: 400 μm. GG: gelatin-genipin hydrogel; GN: NGF loaded GG; GNT: NGF and TFHL dual-loaded GG; NGF: nerve growth factor; NIR: near infrared; SCI: spinal cord injury; TFHL: total flavonoids from the hawthorn leaves.

***Additional Figure 5:*** *Gait analysis results of SCI rat models treated with 10% GNT.*

Additional Figure 5 Gait analysis results of SCI rat models treated with 10% GNT.
The groups were: SCI rats with PBS injection (SCI group), 10% GG injection (10% GG), 10% GN injection (10% GN), 10% GNT injection (10% GNT), and 10% GNT injection combined with NIR irradiation (10% GNT + NIR). In the SCI and 10% GG groups, the rat hind limbs dragged, and rats in the 10% GN group occasionally touched the ground. By contrast, rats in the 10% GNT and 10% GNT + NIR groups touched their hind limbs more. Furthermore, rats in the SCI and 10% GG groups were not as well coordinated as rats in the 10% GN group, and rats in the 10% GNT and 10% GNT + NIR groups were significantly more coordinated than rats in the 10% GN group. GG: Gelatin-genipin hydrogel; GN: NGF loaded GG; GNT: NGF and TFHL dual-loaded GG; LF: left foot; LH: left hand; NGF: nerve growth factor; NIR: near infrared; RF: right foot; RH: right hand; SCI: spinal cord injury; TFHL: total flavonoids from the hawthorn leaves.

***Additional Figure 6:*** *Olig2 and VEGF expression levels in the spinal cord of SCI rat models treated with 10% GNT.*

Additional Figure 6 **OLIG2 and VEGF expression levels in the spinal cord of SCI rat models treated with 10% GNT.**
(A) Immunofluorescent images of OLIG2 expression in the spinal cords of treated rats, and the corresponding MFI of OLIG2 expression (B). (C) Immunofluorescent images of VEGF expression in the spinal cords of treated rats, and the corresponding MFI of VEGF expression (D). The groups were: SCI rats with PBS injection (SCI group), 10% GG injection (10% GG), 10% GN injection (10% GN), 10% GNT injection (10% GNT), and 10% GNT injection combined with NIR irradiation (10% GNT + NIR). Scale bars: 1000 μm and 100 μm. Data are expressed as the mean ± standard deviation (*n* = 6 independent experiments). *P < 0.05, **P < 0.01, ***P < 0.001, ****P < 0.0001 (one-way analysis of variance followed by Tukey's *post hoc* test). DAPI: 4ʹ, 6-Diamidino-2-phenylindole; GG: gelatin-genipin hydrogel; GN: NGF loaded GG; GNT: NGF and TFHL dual-loaded GG; MFI: mean fluorescent intensity; NGF: nerve growth factor; NIR: near infrared; Olig2: oligodendrocyte transcription factor 2; SCI: spinal cord injury; TFHL: total flavonoids from the hawthorn leaves; VEGF: vascular endothelial growth factor.

***Additional Table 1:*** *Primer sequences for reverse transcription-quantitative polymerase chain reaction analysis.*

## Data Availability

*All data relevant to the study are included in the article or uploaded as Additional files*.
