# Supplementary figures and images for: Total flavonoid– and nerve growth factor–loaded gelatin–genipin hydrogel improves repair after spinal cord injury
Source: Neural Regen Res. 2025 Aug 13;21(7):3122–9. doi: 10.4103/NRR.NRR-D-24-01445 (PMC13378959; doi:10.4103/NRR.NRR-D-24-01445)

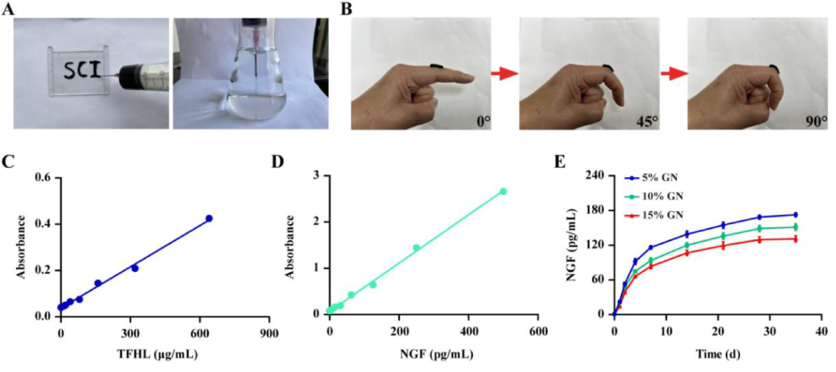

Supplement: Supplementary file 1 [file NRR-21-3122_Suppl1.tif]

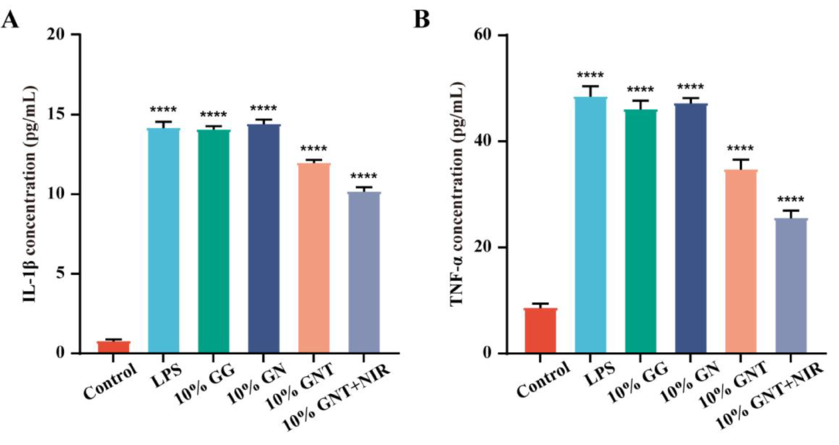

Supplement: Supplementary file 2 [file NRR-21-3122_Suppl2.tif]

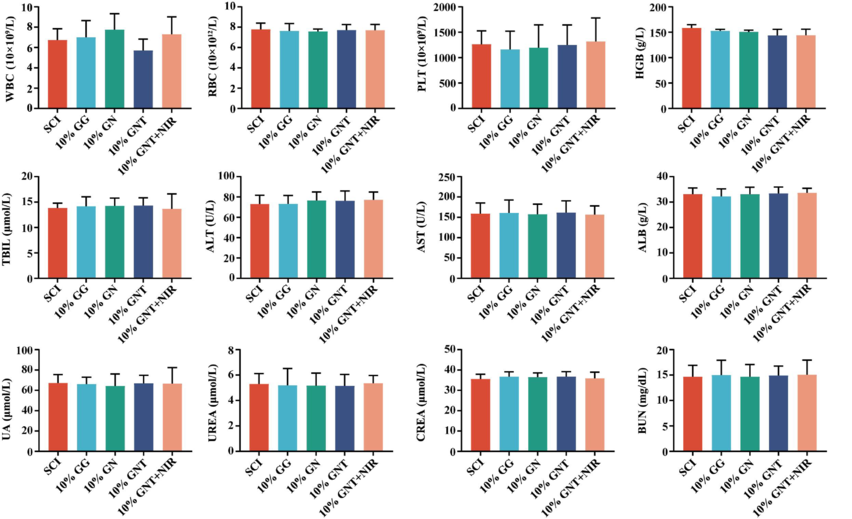

Supplement: Supplementary file 3 [file NRR-21-3122_Suppl3.tif]

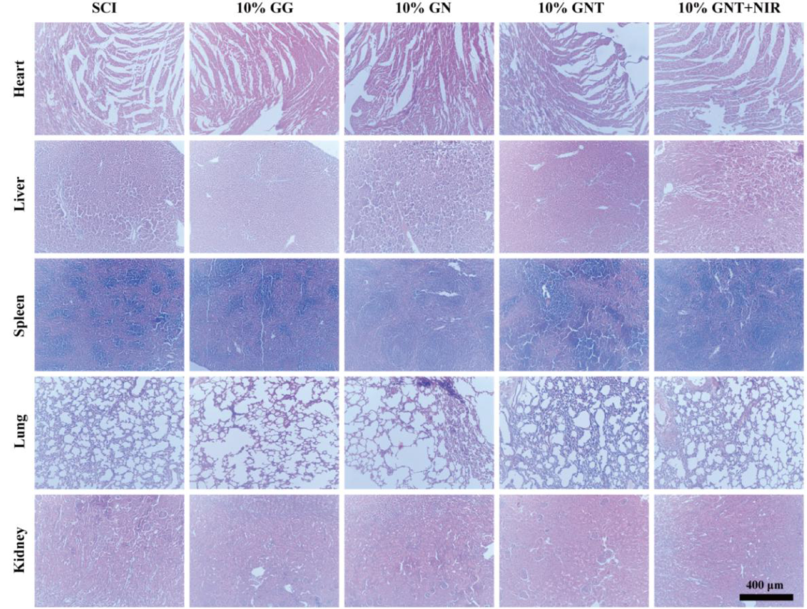

Supplement: Supplementary file 4 [file NRR-21-3122_Suppl4.tif]

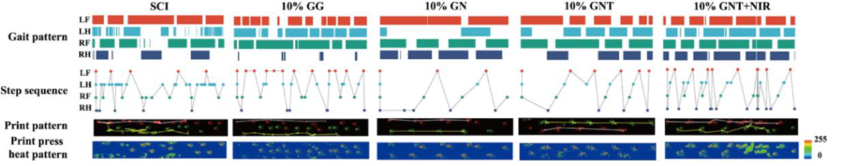

Supplement: Supplementary file 5 [file NRR-21-3122_Suppl5.tif]

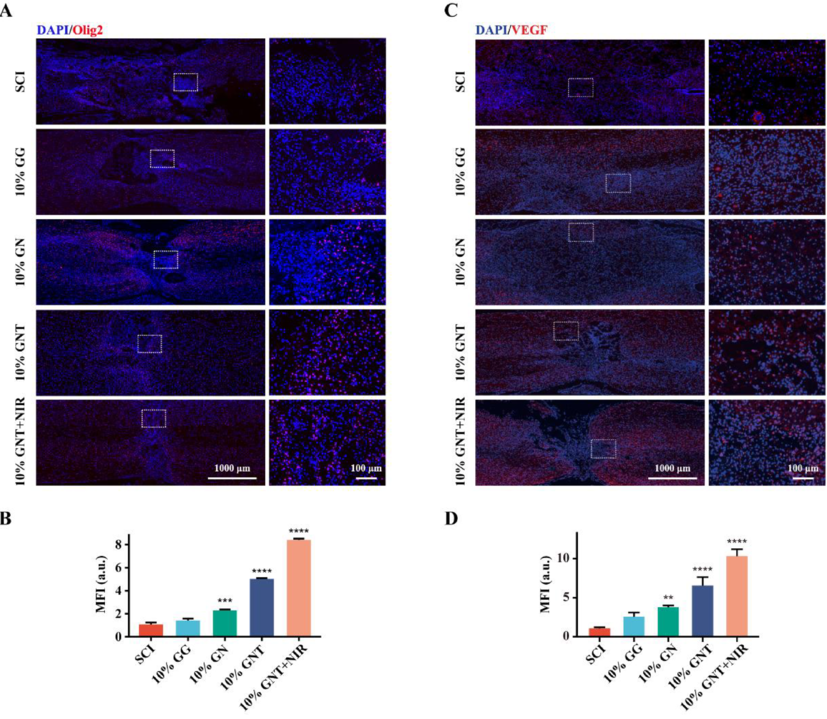

Supplement: Supplementary file 6 [file NRR-21-3122_Suppl6.tif]
